# Supplementary material for: New methods of removing debris and high-throughput counting of cyst nematode eggs extracted from field soil
Source: PLoS One. 2019 Oct 15;14(10):e0223386. doi: 10.1371/journal.pone.0223386 (PMC6793949; doi:10.1371/journal.pone.0223386)
Supplement: S1 Appendix — (DOCX) [file pone.0223386.s001.docx]

# **S1 Appendix. Details of deep learning model.**

Below is a table showing the network parameters of the deep learning model.

**Table 1. Network parameters for the deep learning model.**

| **Layer** | **Filters** | **Parameters** | **Stride** | **Input** | **Output** |
| --- | --- | --- | --- | --- | --- |
| Input | 0 | 0 | 0 | (128, 128, 3) | (128, 128, 3) |
| conv2d_1 | 32 | 896 | (1,1) | (128, 128, 3) | (128, 128, 32) |
| conv2d_2 | 32 | 9248 | (1,1) | (128, 128, 32) | (128, 128, 32) |
| max_pooling2d_1 | 0 | 0 | (2,2) | (128, 128, 32) | (64, 64, 32) |
| conv2d_3 | 64 | 18496 | (1,1) | (64, 64, 32) | (64, 64, 64) |
| conv2d_4 | 64 | 36928 | (1,1) | (64, 64, 64) | (64, 64, 64) |
| max_pooling2d_2 | 0 | 0 | (2,2) | (64, 64, 64) | (32, 32, 64) |
| conv2d_5 | 128 | 73856 | (1,1) | (32, 32, 64) | (32, 32, 128) |
| conv2d_6 | 128 | 147584 | (1,1) | (32, 32, 128) | (32, 32, 128) |
| max_pooling2d_3 | 0 | 0 | (2,2) | (32, 32, 128) | (16, 16, 128) |
| dropout_1 | 0 | 0 | 0 | (16, 16, 128) | (16, 16, 128) |
| conv2d_7 | 256 | 295168 | (1,1) | (16, 16, 128) | (16, 16, 256) |
| conv2d_8 | 256 | 590080 | (1,1) | (16, 16, 256) | (16, 16, 256) |
| max_pooling2d_4 | 0 | 0 | (2,2) | (16, 16, 256) | (8, 8, 256) |
| dropout_2 | 0 | 0 | 0 | (8, 8, 256) | (8, 8, 256) |
| conv2d_9 | 512 | 1180160 | (1,1) | (8, 8, 256) | (8, 8, 512) |
| conv2d_10 | 512 | 2359808 | (1,1) | (8, 8, 512) | (8, 8, 512) |
| dropout_3 | 0 | 0 | 0 | (8, 8, 512) | (8, 8, 512) |
| conv2d_transpose_1 | 256 | 524544 | (2,2) | (8, 8, 512) | (16, 16, 256) |
| concatenate_1 | 0 | 0 | 0 | (16, 16, 512) | (16, 16, 512) |
| conv2d_11 | 256 | 1179904 | (1,1) | (16, 16, 512) | (16, 16, 256) |
| conv2d_12 | 256 | 590080 | (1,1) | (16, 16, 256) | (16, 16, 256) |
| conv2d_transpose_2 | 128 | 131200 | (2,2) | (16, 16, 256) | (32, 32, 128) |
| concatenate_2 | 0 | 0 | 0 | (32, 32, 256) | (32, 32, 256) |
| conv2d_13 | 128 | 295040 | (1,1) | (32, 32, 256) | (32, 32, 128) |
| conv2d_14 | 128 | 147584 | (1,1) | (32, 32, 128) | (32, 32, 128) |
| conv2d_transpose_3 | 64 | 32832 | (2,2) | (32, 32, 128) | (64, 64, 64) |
| concatenate_3 | 0 | 0 | 0 | (64, 64, 128) | (64, 64, 128) |
| conv2d_15 | 64 | 73792 | (1,1) | (64, 64, 128) | (64, 64, 64) |
| conv2d_16 | 64 | 36928 | (1,1) | (64, 64, 64) | (64, 64, 64) |
| conv2d_transpose_4 | 32 | 8224 | (2,2) | (64, 64, 64) | (128, 128, 32) |
| concatenate_4 | 0 | 0 | 0 | (128, 128, 64) | (128, 128, 64) |
| conv2d_17 | 32 | 18464 | (1,1) | (128, 128, 64) | (128, 128, 32) |
| conv2d_18 | 32 | 9248 | (1,1) | (128, 128, 32) | (128, 128, 32) |
| Output | 1 | 33 | (1,1) | (128, 128, 32) | (128, 128, 1) |

**Filter visualizations:**

To begin with, our trained model along with its weights is loaded into memory and the convolutional layers from the encoder path are extracted. An activation loss function is defined to maximize the filter activation. A random noise image of the same size as the input image (128 x 128 x 3) is generated. For each filter, a gradient ascent process is performed for 20 steps starting from the input image with respect to the activation loss function. The resultant filter is post-processed to generate a valid image. For our visualizations, we have taken the best filters which produce the maximum activations in each layer for the input image as shown in Fig 1 to Fig 10 below.

**
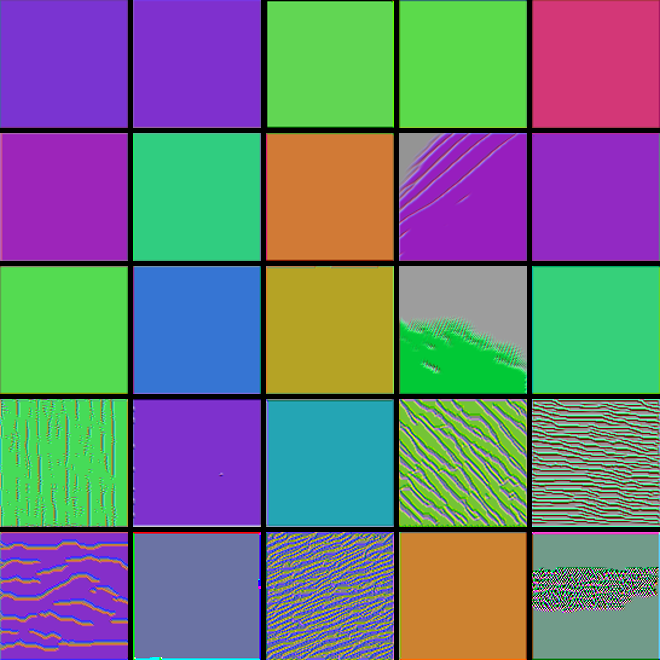
**

**Fig 1. Filters in conv2d_1 that produced maximum activations for a random noise input image.**

**
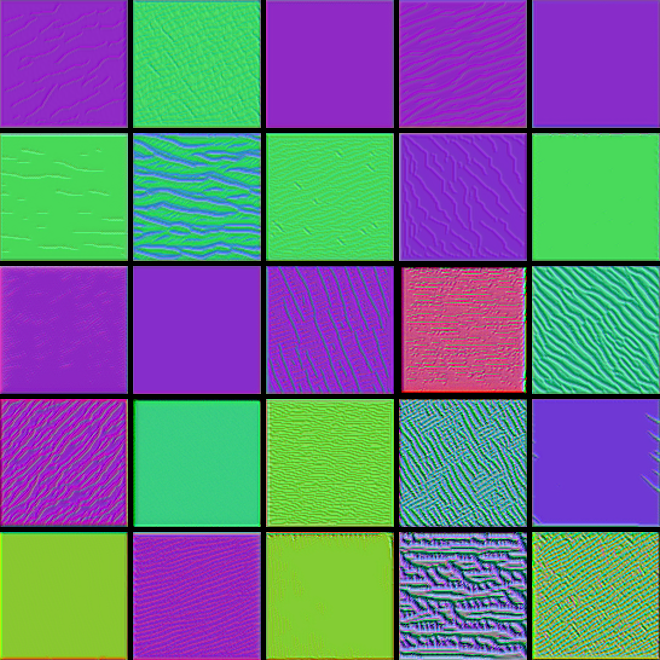
**

**Fig 2. Filters in conv2d_2 that produced maximum activations for a random noise input image.**

**
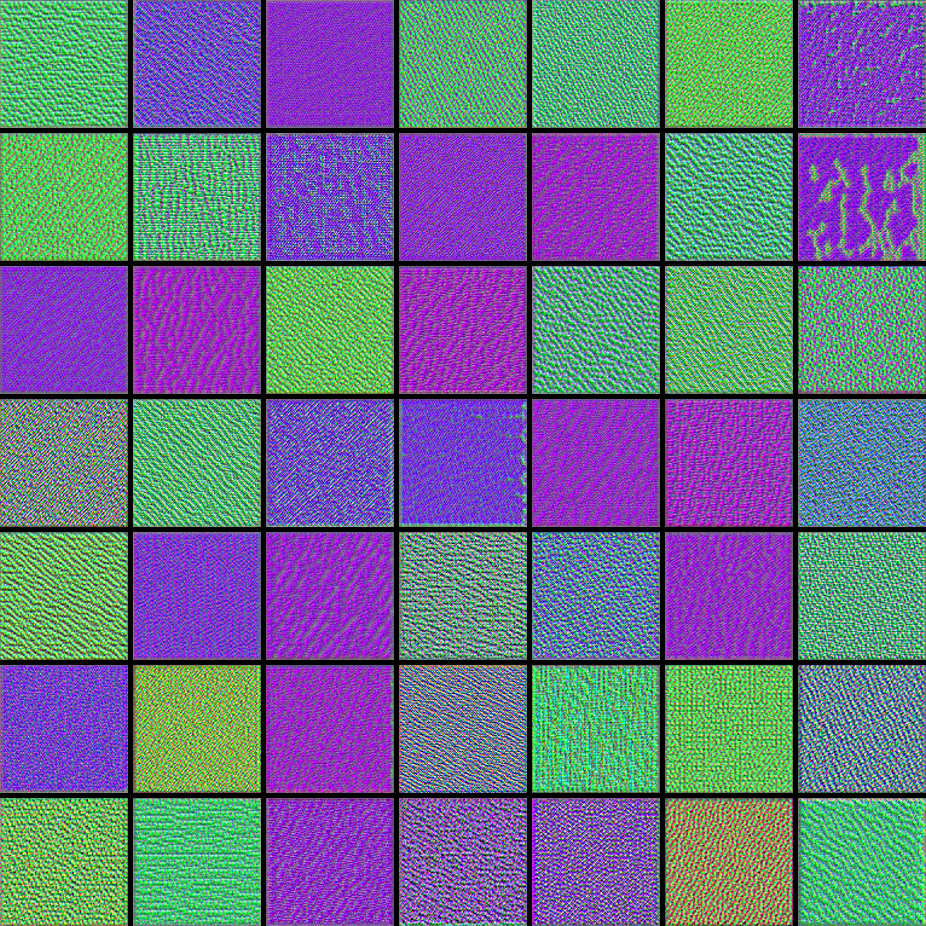
**

**Fig 3. Filters in conv2d_3 that produced maximum activations for a random noise input image.**

**
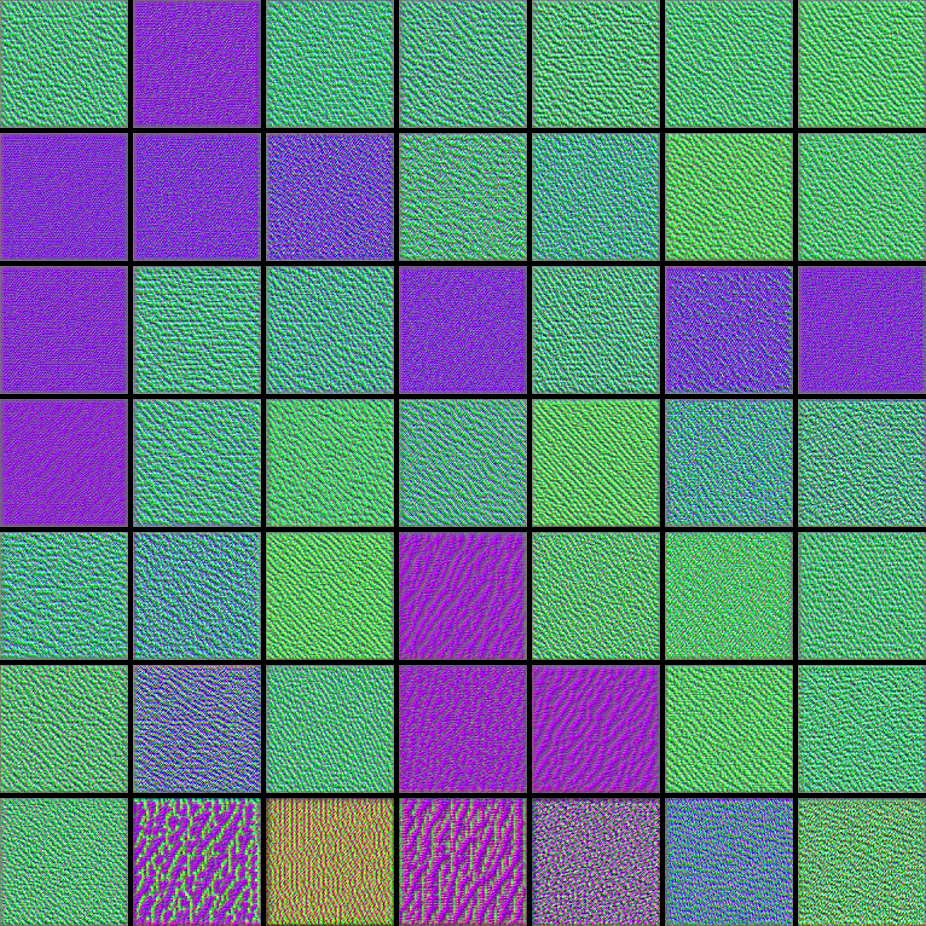
**

**Fig 4. Filters in conv2d_4 that produced maximum activations for a random noise input image.**

**
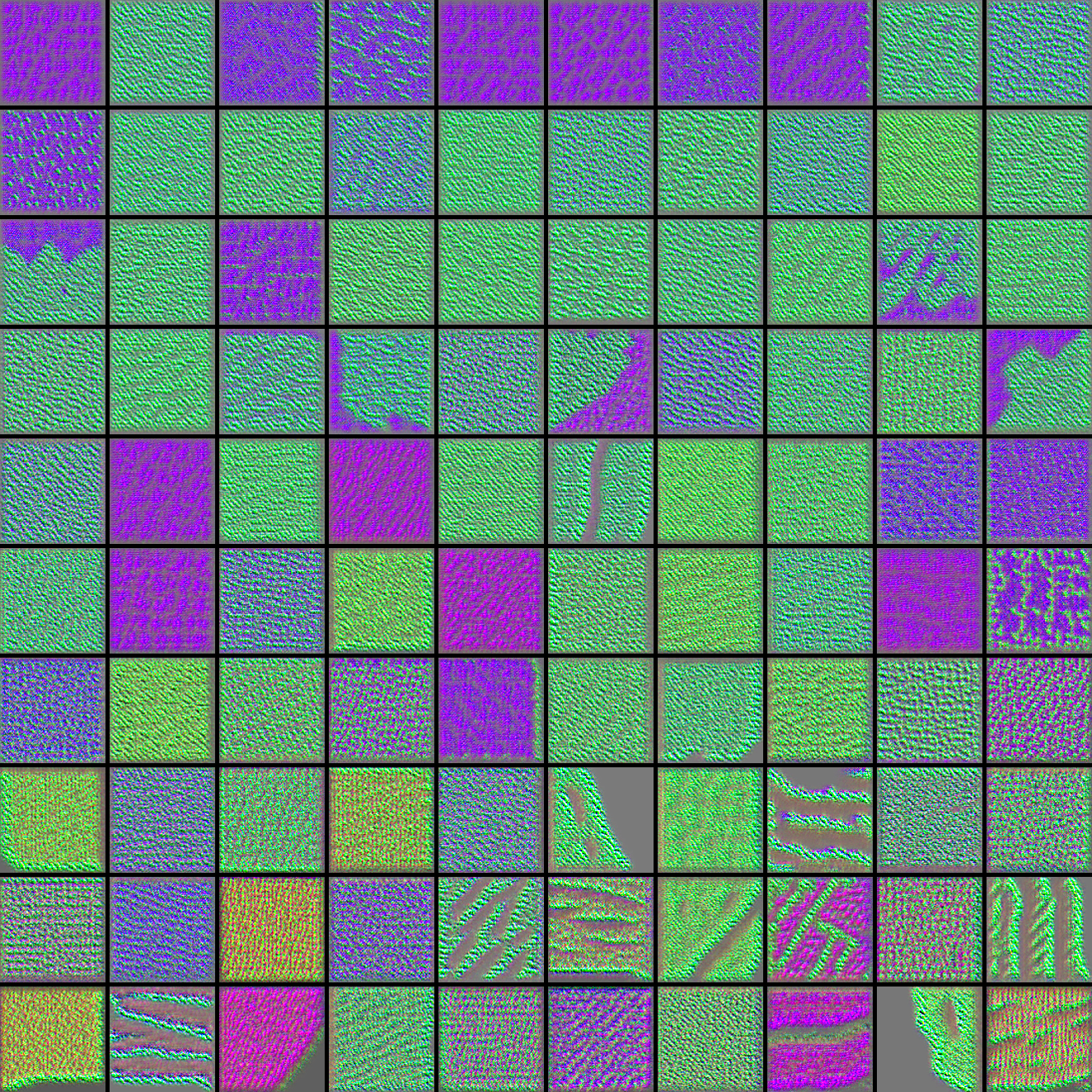
**

**Fig 5. Filters in conv2d_5 that produced maximum activations for a random noise input image.**

**
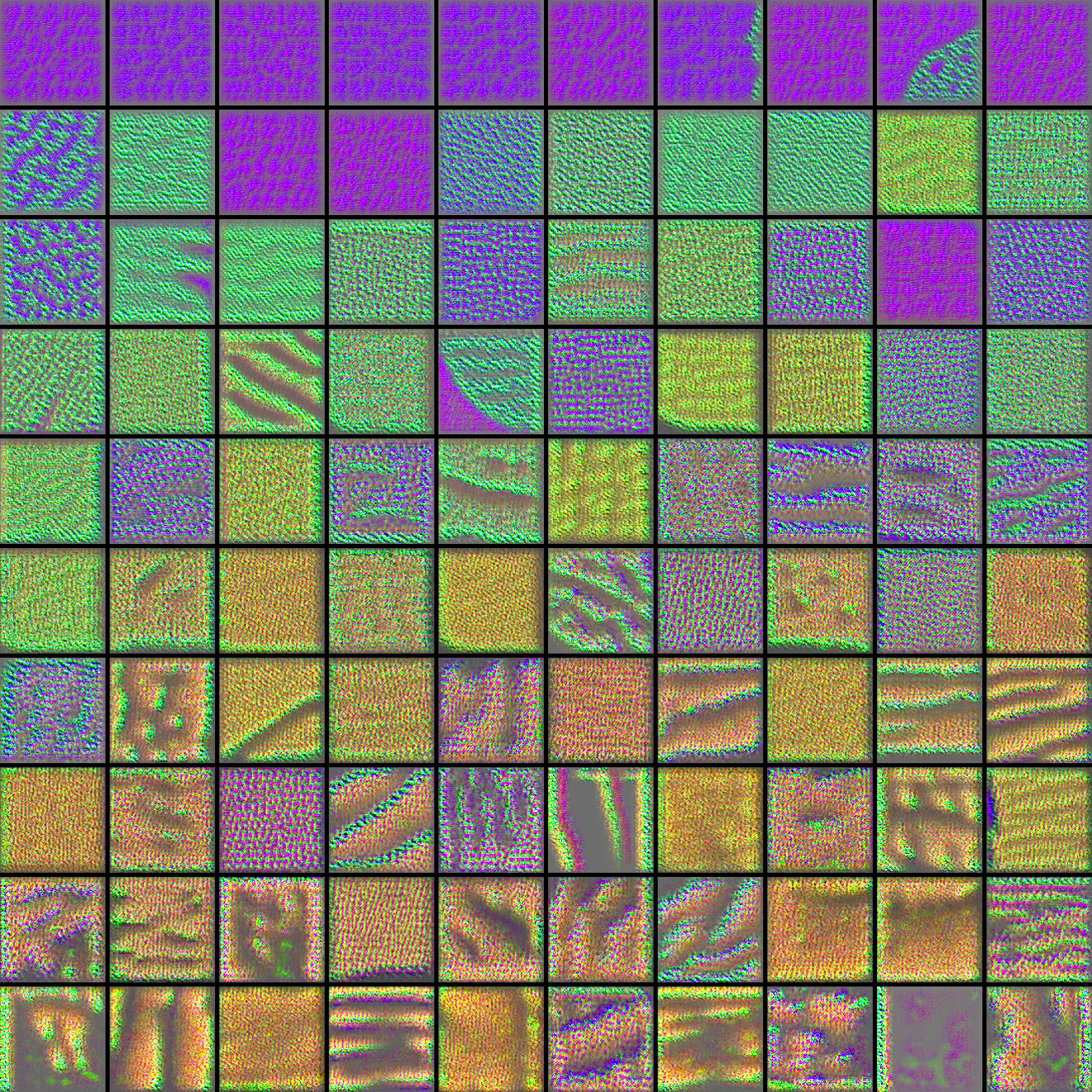
**

**Fig 6. Filters in conv2d_6 that produced maximum activations for a random noise input image.**

**
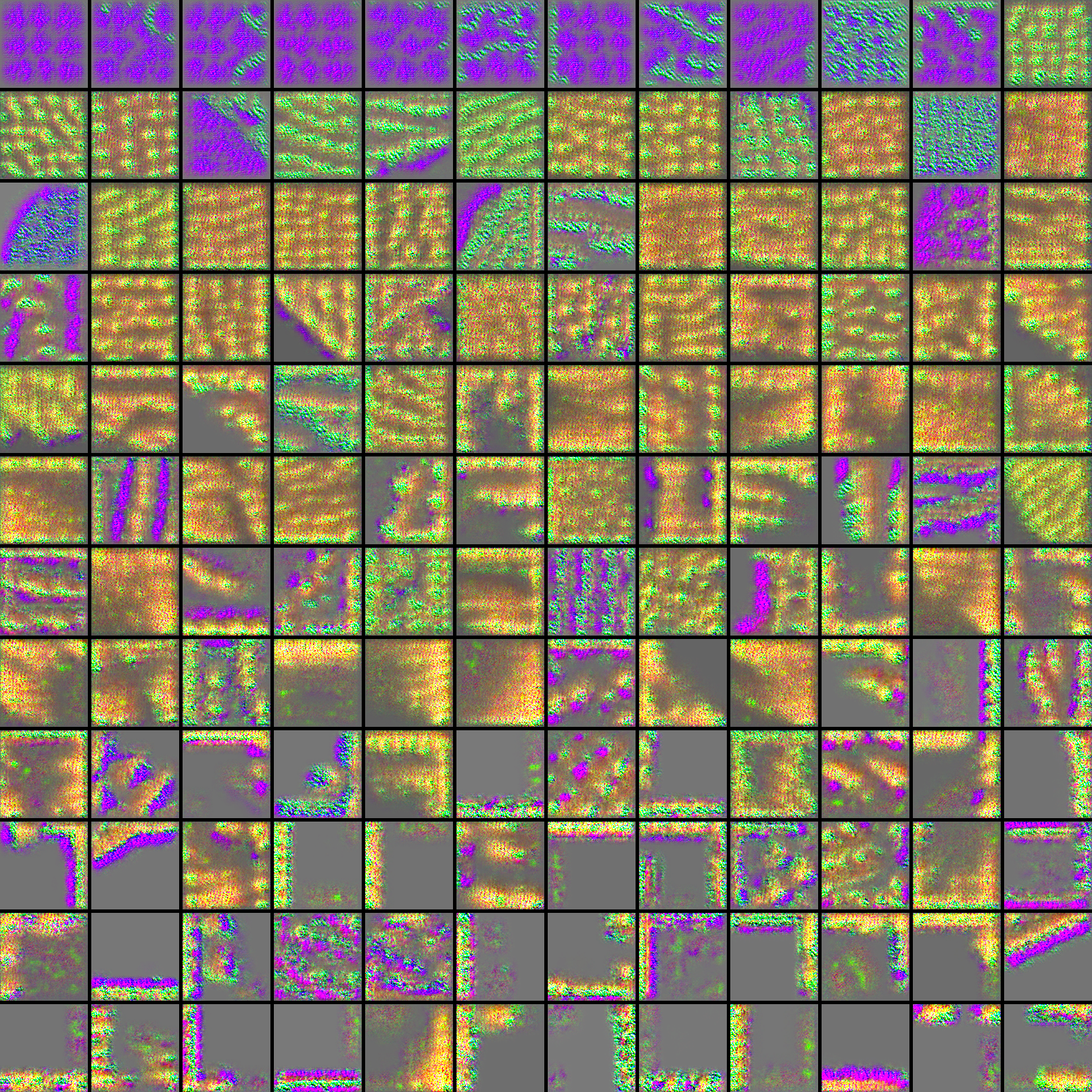
**

**Fig 7. Filters in conv2d_7 that produced maximum activations for a random noise input image.**

**
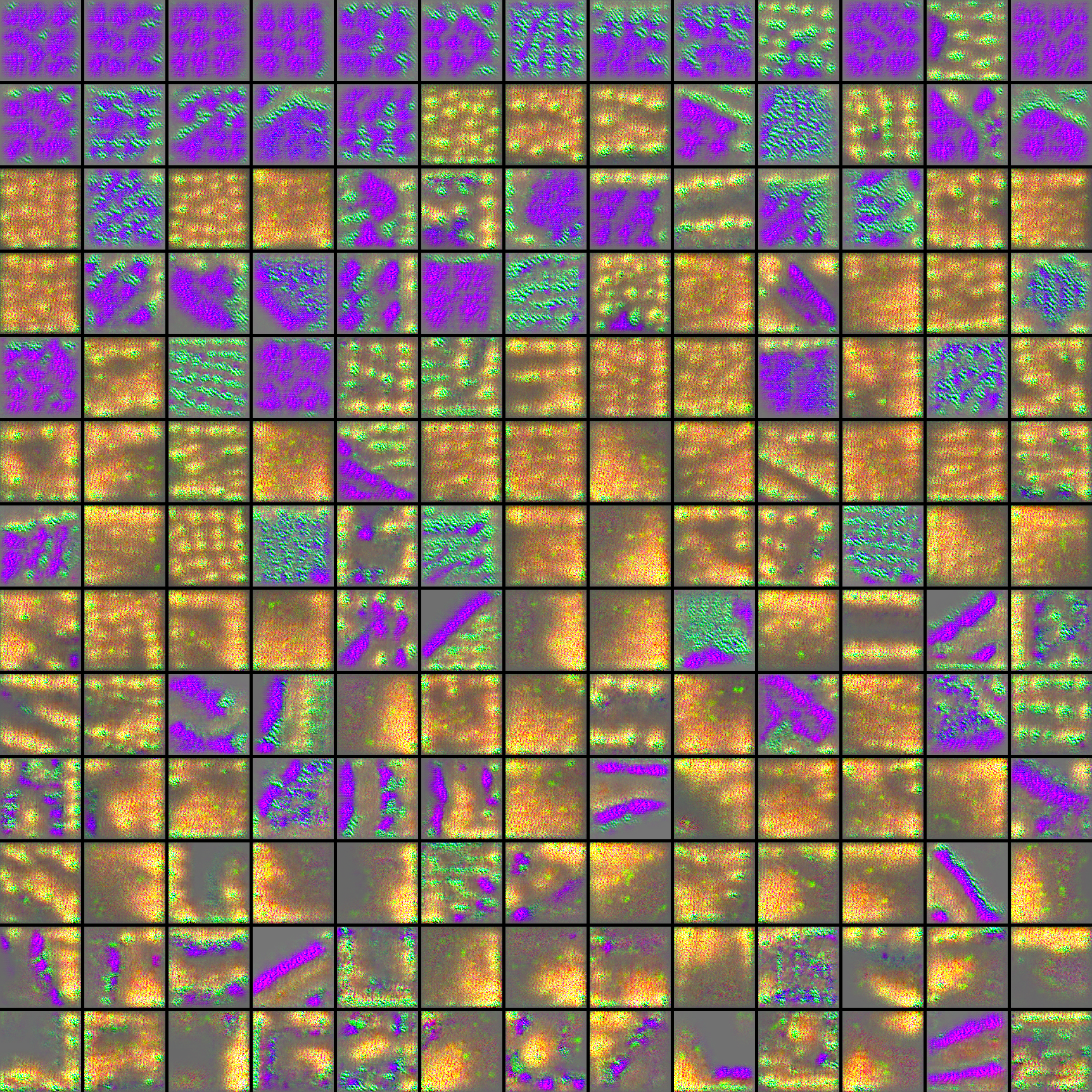
**

**Fig 8. Filters in conv2d_8 that produced maximum activations for a random noise input image.**

**
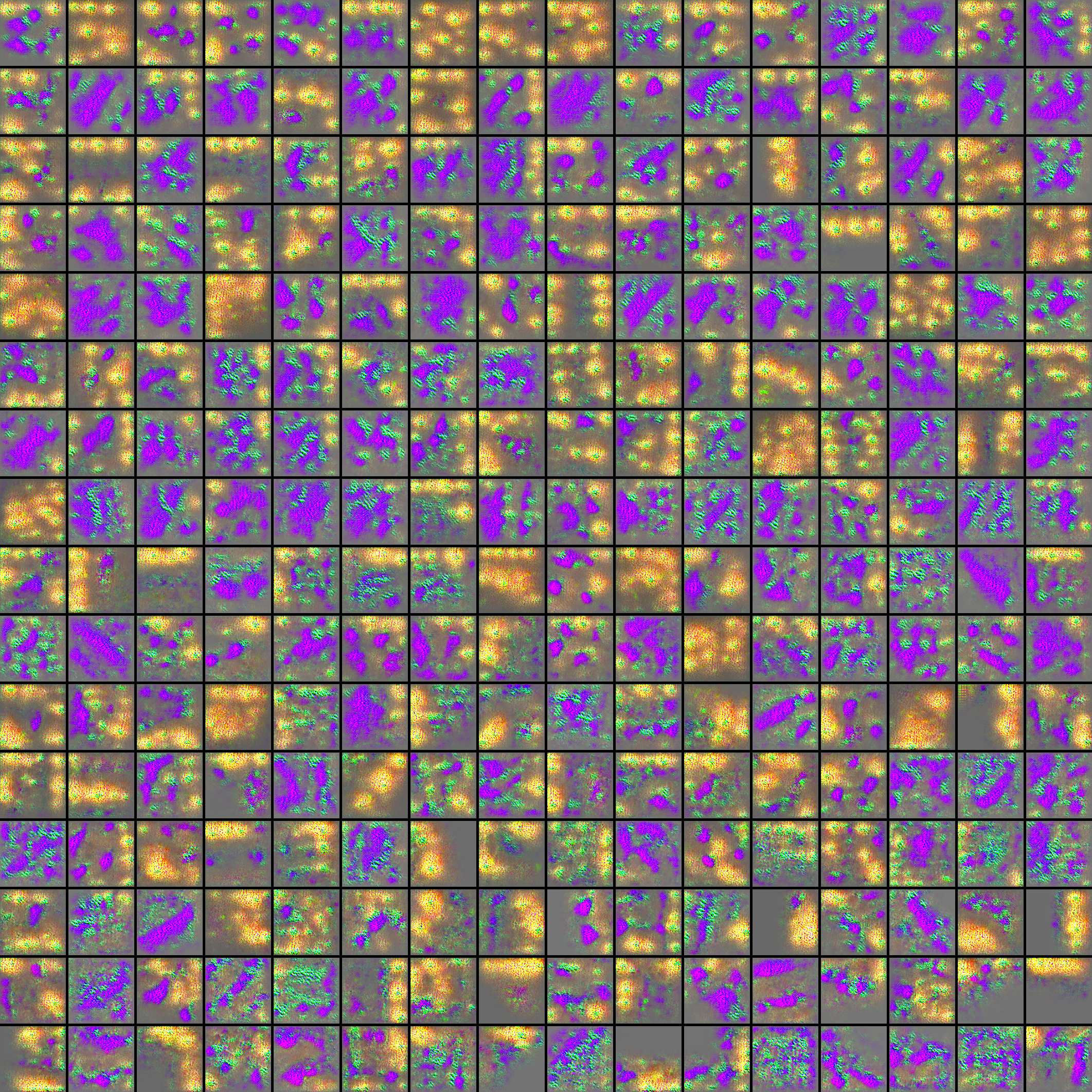
**

**Fig 9. Filters in conv2d_9 that produced maximum activations for a random noise input image.**

**
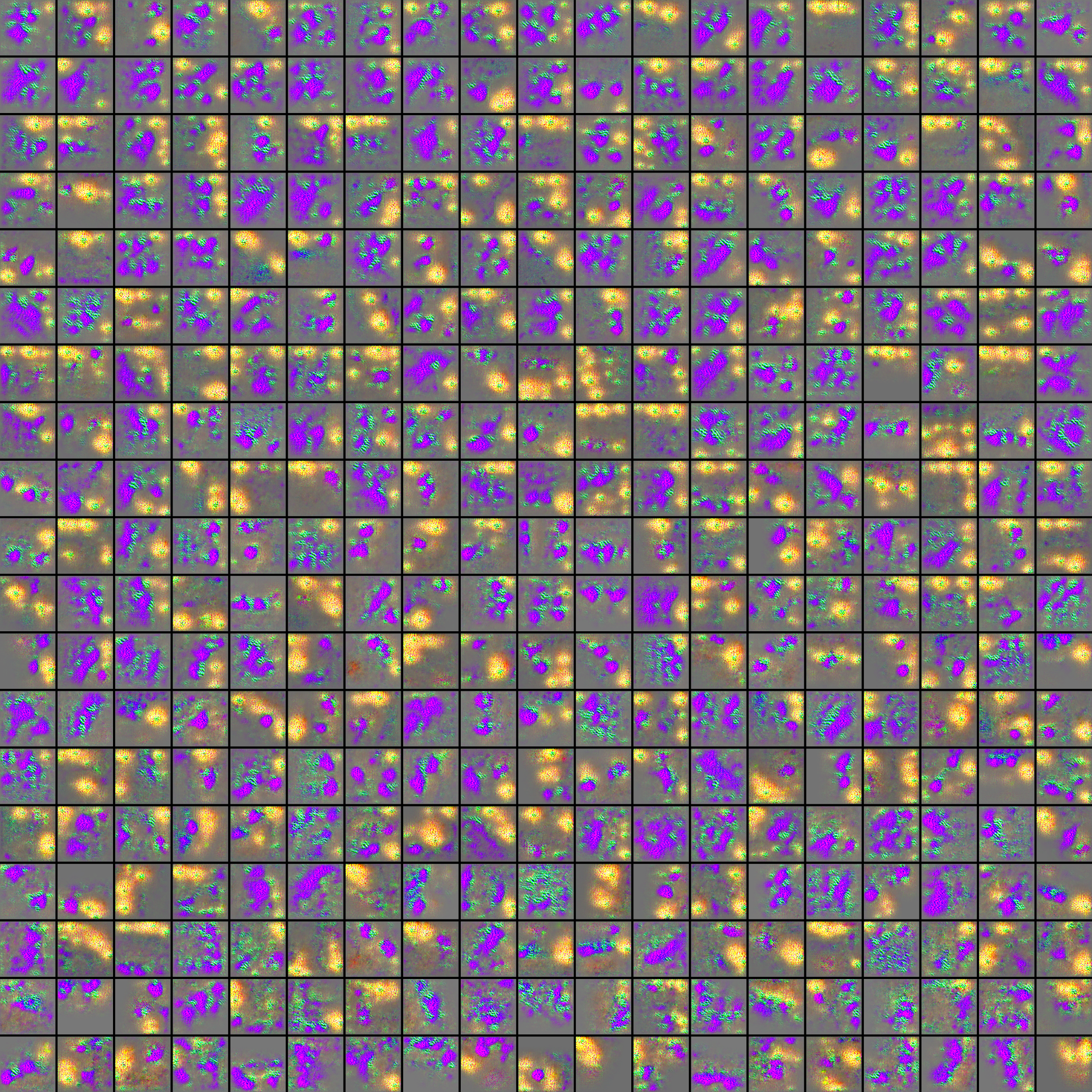
**

**Fig 10. Filters in conv2d_10 that produced maximum activations for a random noise input image.**
